# Commuting Accidents and Sickness Absence Among Health and Social Care Workers in Spain: The Role of Age and Accident Characteristics

**DOI:** 10.3390/healthcare14213709

**Published:** 2026-11-01

**Authors:** Juan Marcos de las Heras, Ignacio Fontaneda, María J. Sierra-Medina, Miguel A. Camino

**Affiliations:** 1 Escuela Politécnica Superior, University of Burgos, 09006 Burgos, Spain; jmdgonzalez@ubu.es; 2 Campus Milanera, University of Burgos, 09001 Burgos, Spain; mjsierra@ubu.es (M.J.S.-M.); macamino@ubu.es (M.A.C.)

**Keywords:** commuting accidents, occupational accidents, healthcare workers, sickness absence, traffic accidents, occupational health, ageing workers

## Abstract

**Highlights:**

**What are the main findings?**

- Sickness absence duration increased with age and was independently longer following traffic-related accidents and accidents occurring on the way from work.
- Shoulder, arm and other upper-limb injuries and wrist injuries showed the largest adjusted injury-site contrasts. The sex association depended on whether injury type and recorded severity were additionally controlled.

**What are the implications of the main findings?**

- Commuting accident prevention in health and social care should consider both accident occurrence and subsequent sickness absence, as the factors associated with these two dimensions may differ.
- Occupational safety strategies should extend beyond the workplace and address commuting risks, with particular attention to older workers, traffic-related accidents, return journeys, and injuries associated with prolonged sickness absence.

**Abstract:**

Background/Objectives: Commuting occupational accidents represent an important but comparatively understudied component of occupational risk among healthcare workers. This study aimed to characterise commuting accidents resulting in sickness absence among workers in the Spanish health and social care sector and to examine associations of age, sex, traffic involvement, commuting direction, and injured body part with sickness absence duration. Methods: This retrospective observational study used secondary administrative data from Spanish occupational accident reports registered through the Delt@ system. We analyzed 139,412 commuting occupational accidents resulting in sickness absence among health and social care workers during 2011–2023. Descriptive and bivariate analyses were complemented by zero-truncated negative binomial regression, with age modelled using a restricted cubic spline and adjustment for sex, traffic involvement, commuting direction, injury site, activity division, workplace province, occupation, source-file year, and employment-contract code. Descriptive and bivariate analyses were conducted using IBM SPSS Statistics version 29. Results: Mean recorded sickness absence duration was 44.15 days. Adjusted mean duration increased non-linearly with age, from 30.59 days at age 25 to 59.24 days at age 65. Traffic involvement and return journeys were associated with longer adjusted mean durations (mean ratios 1.271 and 1.102, respectively). Shoulder, arm and other upper-limb sites and wrist injuries had the largest injury-site contrasts. Sex was not associated with duration in the primary model, but a modest difference emerged after additional adjustment for injury type and severity. Conclusions: Older age, traffic involvement, return journeys, and injury site were associated with recorded sickness absence duration after expanded adjustment. The age association was non-linear, and the sex association depended on clinical adjustment. The findings support considering both accident occurrence and recorded absence burden in commuting prevention.

## 1. Introduction

Commuting occupational accidents, commonly referred to as in itinere accidents, occur during the journey between home and the workplace. Although they take place outside the workplace itself, they constitute an important occupational health problem because commuting is intrinsically linked to participation in work. These accidents include not only road traffic crashes but also non-traffic events, such as falls, slips, or other injuries occurring while walking or using different modes of transport. Previous research in Spain has shown that commuting accidents account for a substantial proportion of occupational injuries and that their patterns differ according to sex and traffic involvement [1]. Beyond their immediate health consequences, commuting accidents can also have longer-term effects on employment, sickness absence, disability, and earnings [2].

Although the immediate causes of commuting accidents occur outside the workplace, employment conditions determine when, how often, and under what circumstances workers are exposed to commuting risks. This perspective may be particularly relevant in the healthcare sector, where work is frequently characterised by high physical demands, repetitive tasks, awkward postures, heavy workloads, shift and night work, and substantial organisational and psychosocial pressures [3,4,5]. Such conditions may affect fatigue, recovery, sleep quality, and alertness before or after work, potentially influencing workers’ ability to commute safely.

Sleep and fatigue are particularly relevant mechanisms linking working conditions with commuting safety. Vargas-Garrido et al. found that sleep problems were associated with commuting accidents rather than workplace accidents, highlighting sleep deprivation as an occupational health issue that extends beyond the workplace [6]. Among nurses, Scott et al. identified associations between work schedules, sleep duration, and drowsy driving, with night work being particularly relevant to difficulties remaining awake while driving home [7]. More recently, HaGani et al. reported associations between burnout, drowsy driving, and commuting crashes among hospital healthcare workers [8]. Research focusing on nurses has also shown that long night shifts may be followed by substantial fatigue and complex post-work sleep and recovery patterns [9]. Similarly, recent evidence published in *Healthcare* indicates that shift work among healthcare professionals is associated with poorer sleep quality and psychological distress [10]. Taken together, these findings suggest that commuting, particularly the journey home after work, may represent an important but insufficiently recognised component of occupational risk among healthcare workers.

Age is another factor that warrants particular attention. Healthcare work combines substantial physical and organisational demands with an ageing workforce. Older nurses may face specific occupational challenges related to physical capacity and demanding working conditions [3]. Recent evidence also suggests that age is associated with work ability among healthcare professionals [11]. In relation to occupational injuries, a previous Spanish study of nursing and nursing assistant professionals found that, once an accident had occurred, increasing age was associated with greater injury severity [12]. However, age may operate differently in relation to accident occurrence and accident consequences. Older workers may have different exposure patterns, experience, or risk behaviours than younger workers, while injuries occurring at older ages may require longer recovery. Consequently, analysing both accident incidence and sickness absence duration can provide complementary information on the occupational health implications of age.

Sex is also relevant when examining commuting occupational accidents. Previous Spanish evidence has identified marked differences between men and women in commuting accident rates and in the relative importance of traffic- and non-traffic-related injuries [1]. This issue is especially important in the health and social care sector because of its predominantly female workforce. Sex differences may reflect not only individual characteristics but also differences in occupational roles, commuting patterns, transport modes, exposure, and injury profiles. Among Spanish nursing workers, sex has also been associated with differences in occupational injury severity [12]. Nevertheless, whether sex remains independently associated with sickness absence duration after accounting for age and accident characteristics is less clear.

Despite their occupational health relevance, commuting accidents among healthcare workers remain comparatively understudied. Much of the research on occupational road safety has traditionally focused on professional drivers and transport-related occupations [13], whereas occupational health research in healthcare workers has concentrated largely on hazards occurring within healthcare facilities. Fadzil and Bulgiba specifically identified commuting accidents among healthcare workers as an understudied occupational health problem [14]. In their study of public hospital nurses, commuting distance was associated with commuting accidents, and the authors also highlighted the possibility that officially reported accidents underestimate their actual occurrence [14]. Similarly, HaGani et al. emphasised commuting crashes and drowsy driving as relevant threats to the safety and well-being of hospital healthcare workers [8]. These findings support the need to extend occupational safety research beyond the boundaries of the healthcare facility itself.

A further research gap concerns the consequences of commuting accidents once they have occurred. Previous studies have primarily examined accident occurrence, associated risk factors, or injury severity, whereas less attention has been paid to sickness absence duration. This outcome provides a complementary indicator of accident burden because it records the duration of the administrative sickness absence episode following an accident. Previous evidence indicates that commuting accidents may have persistent consequences for labour-market participation and sickness absence [2], while research among healthcare workers suggests that personal and occupational characteristics influence the consequences of occupational injuries [12]. Examining sickness absence duration alongside accident incidence may therefore help distinguish factors associated with accident occurrence from those associated with prolonged consequences after an accident.

Accordingly, this study aimed to characterize registered commuting occupational accidents resulting in sickness absence among workers in the Spanish health and social care sector and to examine adjusted associations of age, sex, traffic involvement, commuting direction, and anatomical injury site with recorded sickness absence duration. Accident incidence and sickness absence duration represent complementary but distinct dimensions of occupational health burden: incidence describes accident occurrence relative to the population at risk, whereas duration describes recorded absence following an accident among affected workers. The populations underlying the incidence figures and the individual-level duration analyses are therefore identified separately.

## 2. Materials and Methods

### 2.1. Study Design

This was a retrospective observational study based on the secondary analysis of administrative occupational accident records. The microdata were provided for research purposes by the Spanish Ministry of Labor and Social Economy and originated from occupational accident reports registered through the Delt@ system. The records were collected for administrative reporting rather than specifically for this study. No participants were recruited or contacted. The individual occupational accident was considered the unit of analysis.

### 2.2. Data Source and Study Population

Data were obtained from the Spanish Statistics on Accidents at Work, compiled by the Spanish Ministry of Labour and Social Economy from occupational accident reports registered through the Delt@ system (Electronic Declaration of Injured Workers).

The study included occupational accidents occurring in Spain between 2011 and 2023 among workers employed in the health and social care sector who were registered with the Spanish Social Security system, had occupational contingencies covered, and whose accident resulted in at least one lost working day (LWD).

Occupational accidents in Spain are reported and coded in accordance with the European Statistics on Accidents at Work (ESAW) methodology developed by Eurostat. ESAW establishes harmonised criteria for the collection, coding, and processing of occupational accident data across European Union Member States, thereby enhancing the comparability of occupational accident statistics between countries (Eurostat, 2013).

The source dataset for 2011–2023 contained 7,695,970 occupational accident and relapse records across all economic sectors in Spain. The study population was restricted to workers employed in three economic activities according to the Spanish National Classification of Economic Activities (CNAE-2009), which aligns with NACE Rev. 2: human health activities (Division 86), residential care activities (Division 87), and social work activities without accommodation (Division 88). These activities accounted for 746,556 records, including accidents and relapses.

Within these activities, 144,938 records were classified as occurring “on the way to or from work” in the Place of accident variable and were therefore identified as commuting (in itinere) records. Of these, 5526 were relapses related to previous occupational accidents and were excluded, leaving a final study sample of 139,412 commuting occupational accidents. No records were excluded because of missing data or inconsistent commuting-direction codes.

Only occupational accidents resulting in at least one day of sickness absence were eligible for inclusion. Accidents without sickness absence were outside the study population. Consequently, the outcome had a minimum value of one day by design, and all sickness absence duration analyses were conditional on an accident having resulted in sickness absence.

Commuting direction was derived from the Hora de trabajo field, labelled Hours worked in the study dataset, following the official Delt@ instructions for completing the occupational accident report. For commuting accidents, code 0 indicates the journey to work, whereas code 99 indicates the return journey home. These values are special administrative codes rather than actual numbers of hours worked [15]. All selected accident records contained one of these two valid codes.

### 2.3. Study Variables

The outcome variable was sickness absence duration, measured as the number of days lost following each commuting occupational accident. In accordance with the European Statistics on Accidents at Work (ESAW) terminology, this information corresponds to the variable Days lost, which reflects the consequences of the accident in terms of absence from work. Recorded sickness absence duration was used as an indicator of absence-related burden following an accident. It should not be interpreted as a direct measure of injury severity or as verified time to recovery or return to work. Duration may also reflect functional limitations, job demands, treatment and rehabilitation, administrative procedures, and return-to-work arrangements.

The explanatory variables included characteristics of the injured worker and of the accident. Worker characteristics comprised age and sex, corresponding to the ESAW variables Age of the victim and Sex of the victim. Age was treated as a continuous quantitative variable, whereas sex was analysed as a categorical variable (male/female).

Accident-related variables included traffic involvement, commuting direction, and part of body injured. Accidents were classified according to whether they were traffic-related or non-traffic-related. Commuting direction distinguished accidents occurring on the way to work from those occurring on the way from work.

The anatomical location of the injury was analysed using the ESAW variable Part of body injured. ESAW codes this variable using a two-digit classification identifying the anatomical region affected by the accident. For the present analysis, the original categories were consolidated into broader anatomically homogeneous groups to facilitate statistical analysis and interpretation.

Additional adjustment variables were activity division (CNAE2digitos), workplace province (PROVCEN), three-digit occupational category (CNO2011), year (AÑO), and employment-contract code (TIPCONT). Injury type (DLESION) and recorded injury severity (GLESION) were considered in an additional adjustment model. Detailed diagnostic codes (DIAG) were incompletely recorded and were not included in the primary analysis.

The selection and grouping of variables were based on their relevance to the study objectives and their availability in the Spanish occupational accident database. The dependent and explanatory variables therefore captured three complementary dimensions: the consequences of the accident in terms of sickness absence duration, individual characteristics of the injured worker (*age and sex*), and characteristics of the commuting accident itself (*traffic involvement, commuting direction, and part of body injured*). Additional information on the coding of traffic involvement and anatomical injury sites is provided in Appendix A.

The maximum recorded sickness absence duration in the study dataset was 545 days. No additional upper limit was imposed by the researchers. This value coincides with the exhaustion threshold for temporary incapacity under the Spanish Social Security framework; however, reaching this threshold does not necessarily indicate recovery or return to work, and the economic effects of temporary incapacity may continue beyond it in specified circumstances [16]. The available data did not establish whether records at 545 days represented completed absence episodes, administrative closure, or ongoing absence. These observations were therefore interpreted as recorded administrative durations rather than verified times to return to work.

### 2.4. Statistical Analysis

Descriptive analyses were performed to characterise the study sample and sickness absence duration. Categorical variables were summarised as absolute and relative frequencies [n (%)]. Sickness absence duration was described using the mean and standard deviation (SD), median and interquartile range (P25–P75), and minimum and maximum values. Skewness and kurtosis were also calculated to assess the shape of its distribution.

**Figure 1:**
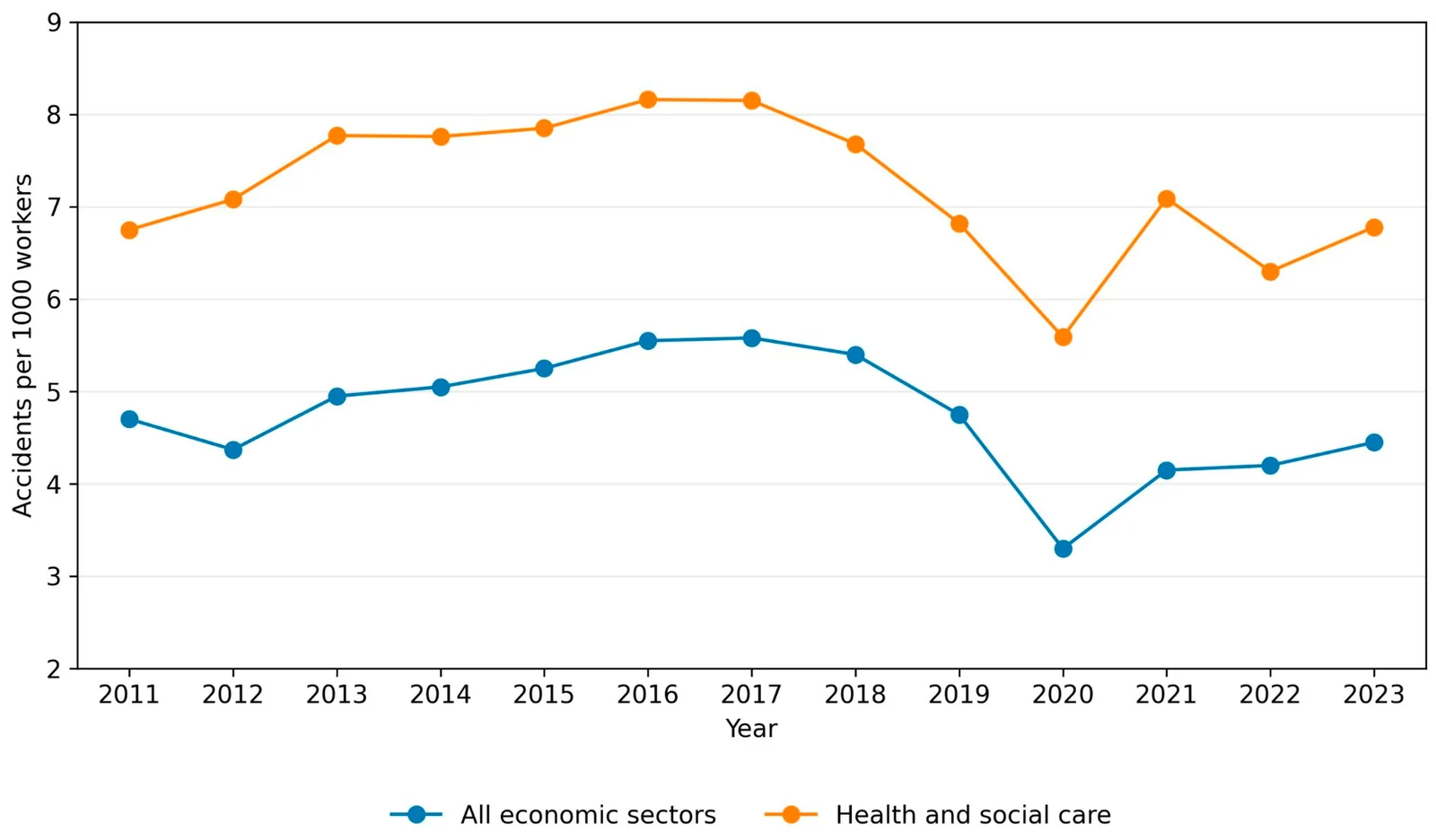
Incidence rates of commuting occupational accidents resulting in sickness absence in Spain: comparison between the health and social care sector and all economic sectors combined, 2011–2023 (accidents per 1000 workers).

**Figure 2:**
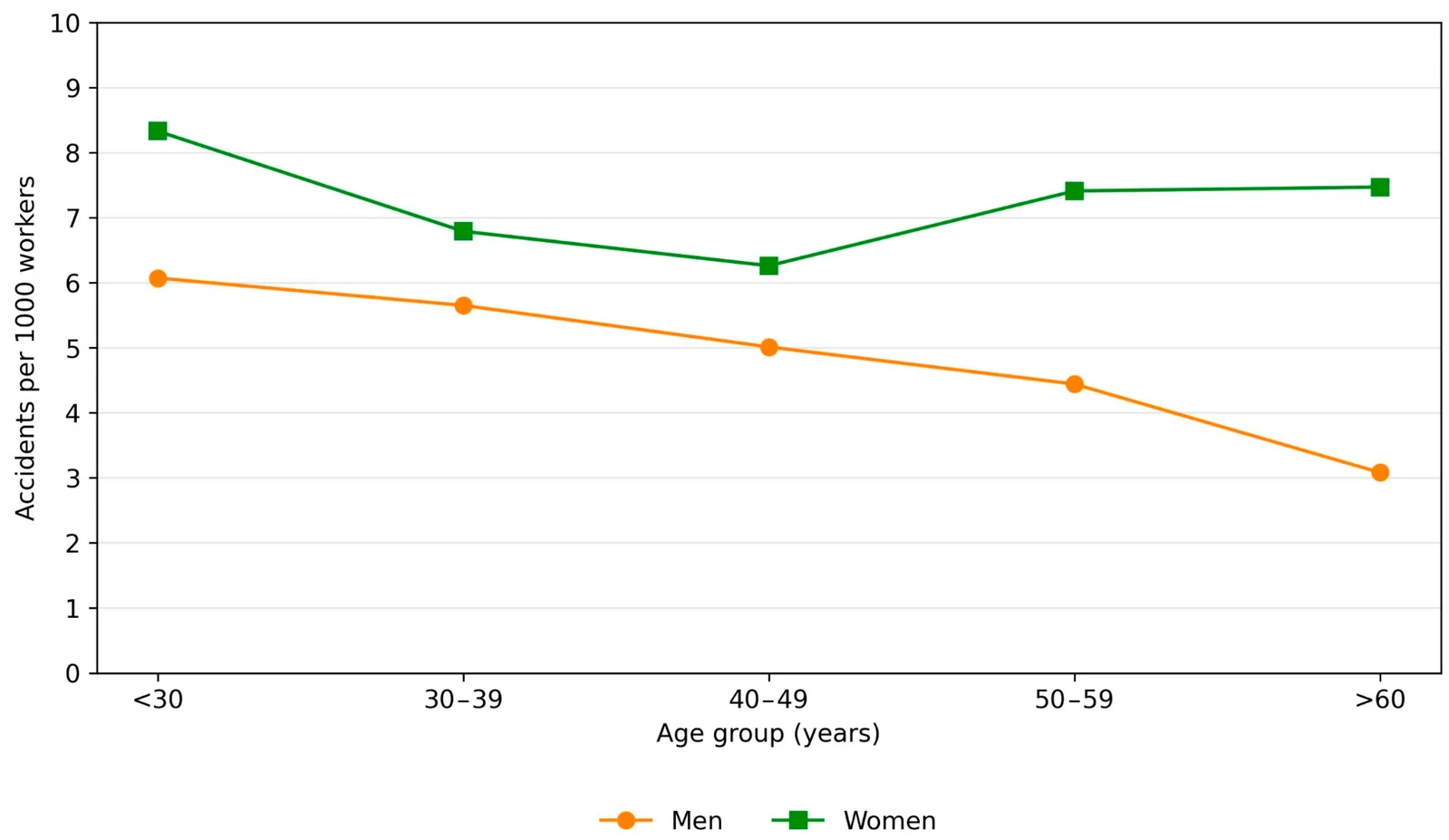
Incidence rates (accidents per 1000 workers) of commuting occupational accidents resulting in sickness absence in Spain (health and social care sector), by age group and sex, 2023.

Incidence rates were expressed as commuting occupational accidents resulting in sickness absence per 1000 workers affiliated with the Spanish Social Security system and covered for occupational contingencies. Figure 1 compares health and social care activities with all economic sectors during 2011–2023; Figure 2 presents health and social care rates by age group and sex in 2023. Numerators and denominators corresponded to the same population and period for each estimate, with additional age and sex stratification for Figure 2.

Associations between categorical variables were assessed using Pearson’s chi-square (χ^2^) test. The distribution of commuting occupational accidents across age groups was compared according to sex, commuting direction (on the way to work vs. on the way from work), and traffic involvement (traffic-related vs. non-traffic-related accidents). Differences in mean sickness absence duration between two independent groups were assessed using Student’s *t*-test within each age group. Comparisons were performed between men and women, accidents occurring on the way to and from work, and traffic-related and non-traffic-related accidents. In addition, the distribution of injured body parts and mean sickness absence duration were examined descriptively according to sex and traffic involvement.

The regression analysis aimed to estimate adjusted associations with recorded sickness absence duration among accident cases with positive absence, rather than to develop an individually validated prediction model. Ordinary and zero-truncated negative binomial models were fitted using the NB2 parameterisation. The latent negative binomial distribution had mean parameter μ and variance μ + αμ^2^, with a log link and an estimated dispersion parameter α. For the zero-truncated model, the likelihood was conditional on a positive duration. All 139,412 selected accidents were included in the primary regression.

The primary model included age, sex, traffic involvement, commuting direction, injured body part, activity division (CNAE-2009 divisions 86–88), workplace province (PROVCEN), occupational category (three-digit CNO-2011), year (Año), and employment-contract code (TIPCONT). All variables except age were represented by categorical indicator variables. Missing contract information in 78 records was retained as a separate unknown category; the recorded contract code 0 was retained separately. Reference categories were women, non-traffic-related accidents, journeys to work, head injuries, activity division 86, province code 28, occupational code 561, source-file year 2011, and contract code 100.

Age was modelled using a restricted cubic spline with four knots at the 5th, 35th, 65th, and 95th percentiles of the observed age distribution (24, 39, 51, and 62 years), allowing a non-linear association on the log-mean-parameter scale [17]. Departure from a linear age term was assessed using a joint robust Wald test of the two non-linear spline coefficients. Model-based adjusted mean durations were calculated at ages 25, 35, 45, 55, and 65 years by averaging the positive-duration conditional means over the observed distribution of all other covariates.

For the zero-truncated model, the expected positive duration was calculated as μ/[1 − P(Y = 0)]. Adjusted mean ratios were obtained by setting the characteristic of interest to each comparison category in turn, retaining the observed values of the remaining covariates, and taking the ratio of the averaged conditional means. These marginally standardised ratios were not equated with exponentiated coefficients of the latent count model. Confidence intervals and tests used an observation-level sandwich covariance estimator and the delta method.

Model assessment included the estimated dispersion parameter, log likelihood, Akaike and Bayesian information criteria, the Pearson residual statistic relative to residual degrees of freedom, calibration by deciles of fitted duration, randomized quantile residuals [18], numerical convergence, and case-influence diagnostics. Sensitivity analyses used an ordinary NB2 model with the same covariates and spline, excluded the 34 records at 545 days, excluded the 117 fatal accidents, replaced annual source-file year by recorded accident year, used broad CNO-2011 occupational groups, and excluded the 0.1% of records with the largest generalised case-influence scores. An additional model included injury type (DLESION) and recorded injury severity (GLESION). Detailed diagnosis codes (DIAG) were not included because 40,045 records lacked usable diagnostic information. A time-to-return-to-work analysis was not undertaken because a verified return-to-work event and censoring status could not be reconstructed from the supplied duration field.

Descriptive and bivariate analyses were conducted using IBM SPSS Statistics, version 29. The revised likelihood-based regression analyses, marginal standardisation, sandwich covariance estimation, and diagnostics were implemented in Python 3.12 using NumPy 2.3.5 and SciPy 1.17.0 [19]. Statistical tests were two-sided.

## 3. Results

### 3.1. Descriptive Analysis

A total of 139,412 commuting occupational accidents were included in the analysis. Recorded sickness absence duration ranged from 1 to 545 days, with a mean of 44.15 days (SD = 55.97). A total of 34 accidents (0.0244%) had a recorded duration of exactly 545 days. The distribution was markedly right-skewed (skewness = 3.09) and leptokurtic (kurtosis = 13.23), indicating that most accidents resulted in relatively shorter periods of sickness absence, while a smaller proportion were associated with substantially longer durations.

Table 1 presents the characteristics of the study sample and sickness absence duration according to sex, commuting direction, traffic involvement, and injured body part. Women accounted for the majority of commuting accidents (82.6%), whereas men represented 17.4%. Median sickness absence duration was similar for men and women (25 days), although the mean was slightly higher among men (45.84 vs. 43.80 days).

Most accidents occurred on the way to work (70.2%), compared with 29.8% on the way from work. Accidents occurring on the way from work were associated with longer sickness absence, with a median duration of 28 days (P25–P75: 13–59) compared with 24 days (10–51) for accidents occurring on the way to work. Traffic-related accidents represented 46.5% of the sample, while 53.5% were non-traffic-related. Median sickness absence duration was higher for traffic-related accidents (27 vs. 23 days), although their mean duration was slightly lower (43.93 vs. 44.35 days).

Regarding the injured body part, the neck was the most frequently affected anatomical region (24.75%), followed by the ankle (12.7%), multiple body sites (12.27%), and the leg, including the knee (10.3%). Substantial differences in sickness absence duration were observed across anatomical regions. The longest durations corresponded to shoulder and arm injuries, with a median of 44 days (16–98) and a mean of 70.35 days (SD = 75.36), followed by wrist injuries, with a median of 43 days (16–93) and a mean of 65.01 days (SD = 67.41). In contrast, facial injuries showed the shortest sickness absence duration, with a median of 13 days (6–28) and a mean of 25.90 days (SD = 41.88).

### 3.2. Incidence Rates of Commuting Occupational Accidents

Figure 1 shows the evolution of the incidence rates of commuting occupational accidents resulting in sickness absence in Spain from 2011 to 2023, comparing the health and social care sector with all economic sectors combined. Incidence rates were calculated as the number of commuting occupational accidents per 1000 exposed workers affiliated with the Social Security system and covered for occupational contingencies, using data from the Spanish Ministry of Labor and Social Economy. Throughout the period, incidence rates were consistently higher in the health and social care sector than across all economic sectors combined. Both series reached their highest levels in 2016–2017 and subsequently declined, with a marked decrease in 2020, coinciding with the COVID-19 pandemic and restrictions on mobility. Rates increased in both groups in 2021. Thereafter, the overall rate continued to rise, whereas the rate in the health and social care sector decreased in 2022 before increasing again in 2023. In both groups, the rates recorded in 2023 remained below their pre-pandemic levels (Figure 1).

The age-specific incidence rates of commuting occupational accidents resulting in sickness absence among health and social care workers in Spain in 2023 showed different patterns for men and women (Figure 2). Among men, the incidence rate decreased consistently with increasing age, from 6.07 accidents per 1000 workers among those aged under 30 years to 3.08 among those aged over 60 years. Among women, the rate declined from 8.33 accidents per 1000 workers in the youngest age group to 6.26 in the 40–49-year age group, before increasing to 7.41 among those aged 50–59 years and 7.47 among those aged over 60 years. Incidence rates were higher among women in every age group, with the largest absolute difference observed in the oldest group (Figure 2).

### 3.3. Age-Related Patterns by Sex, Commuting Direction, and Traffic Involvement

Table 2 shows the distribution of commuting occupational accidents and mean sickness absence duration according to age and sex. The age distribution differed significantly between men and women (χ^2^ = 839.7, *p* < 0.001). Among men, the highest proportion of accidents occurred in the 40–49-year age group (26.57%), closely followed by those aged 30–39 years (26.26%). In contrast, among women, the largest proportion was observed in the 50–59-year age group (29.80%). Workers aged over 60 years accounted for a higher proportion of accidents among women than among men (10.33% vs. 6.92%).

Sickness absence duration increased progressively with age in both sexes. Men showed a higher mean sickness absence duration than women in every age group, with the difference becoming particularly pronounced in the 40–49 and 50–59-year groups (47.64 vs. 43.27 days and 57.93 vs. 50.98 days, respectively). Differences between men and women were statistically significant in all age groups below 60 years, whereas no significant difference was observed among workers aged over 60 years (59.85 vs. 58.47 days; *p* = 0.451).

Commuting direction also showed a significant association with age (χ^2^ = 31.799, *p* < 0.001), although the age distributions of accidents occurring on the way to and from work were broadly similar (Table 3). Overall, approximately 70% of commuting accidents occurred on the way to work, while around 30% occurred on the way from work. In both commuting directions, the highest proportion of accidents was recorded among workers aged 50–59 years. Despite being less frequent, accidents occurring on the way from work were consistently associated with longer sickness absence duration across all age groups. Mean sickness absence duration ranged from 28.66 to 56.29 days for accidents occurring on the way to work, compared with 33.59 to 64.34 days for those occurring on the way from work. Differences between commuting directions were statistically significant in every age group (all *p* < 0.001). Moreover, sickness absence duration increased steadily with age regardless of commuting direction (Table 3).

A different age pattern emerged when accidents were classified according to traffic involvement (Table 4). The age distribution differed significantly between traffic-related and non-traffic-related accidents (χ^2^ = 12,979, *p* < 0.001). The proportion of non-traffic-related accidents increased markedly with age, from 9.15% among workers aged under 30 years to a maximum of 36.99% among those aged 50–59 years. Conversely, traffic-related accidents were more concentrated in younger age groups, reaching their highest proportion among workers aged 30–39 years (29.17%) and subsequently decreasing with age, particularly among workers aged over 60 years (4.94%).

Across the reported age groups, the unadjusted mean duration was 4.45–8.05 days longer for return-journey accidents than for journeys to work.

Traffic-related accidents were associated with a longer mean sickness absence duration than non-traffic-related accidents in every age group. For example, among workers aged under 30 years, mean sickness absence duration was 31.57 days for traffic-related accidents compared with 27.32 days for non-traffic-related accidents; among those aged over 60 years, the corresponding values were 62.03 and 57.59 days. These differences were statistically significant across all age groups (*p* < 0.001 for workers aged <60 years; *p* = 0.002 for those aged >60 years). As observed for sex and commuting direction, sickness absence duration increased consistently with age in both traffic-related and non-traffic-related accidents (Table 4).

Traffic-related accidents had an unadjusted mean duration 4.25–7.37 days longer than non-traffic-related accidents within the reported age groups, despite their similar overall crude means.

### 3.4. Injured Body Part by Sex and Traffic Involvement

The distribution of injured body parts showed marked differences according to sex and traffic involvement (Table 5). Among non-traffic-related accidents, lower-extremity injuries predominated, particularly among women. Ankle injuries were the most frequent among women, accounting for 23.13% of non-traffic-related accidents, followed by injuries to the leg, including the knee (15.42%), and the foot (12.43%). Among men, ankle (17.16%) and leg, including knee (16.82%), injuries were also the most frequent, although shoulder and arm injuries accounted for a comparatively larger proportion (11.33%).

A markedly different anatomical pattern was observed for traffic-related accidents. Neck injuries were particularly prominent among women, accounting for 54.41% of all traffic-related accidents in women, substantially exceeding the proportion observed among men (29.87%). Multiple body sites represented the second most frequent category among women (15.33%). Among men, although neck injuries were also the most frequent (29.87%), the distribution was more dispersed across anatomical regions, with multiple body sites (21.11%), shoulder and arm (9.43%), back (8.92%), and leg, including knee (7.48%) injuries also accounting for substantial proportions. These findings indicate a clear shift from predominantly lower-extremity injuries in non-traffic-related accidents to neck and multiple-site injuries in traffic-related accidents, particularly among women (Table 5).

Sickness absence duration also varied considerably according to the injured body part, sex, and traffic involvement. Shoulder and arm injuries were consistently associated with some of the longest sickness absence durations. In non-traffic-related accidents, mean sickness absence duration for shoulder and arm injuries was 66.68 days in men and 75.98 days in women. In traffic-related accidents, the corresponding mean durations were 66.00 and 58.13 days, respectively. Wrist injuries were also associated with prolonged sickness absence, particularly in women following non-traffic-related accidents (66.90 days) and traffic-related accidents (65.28 days).

**Figure 3:**
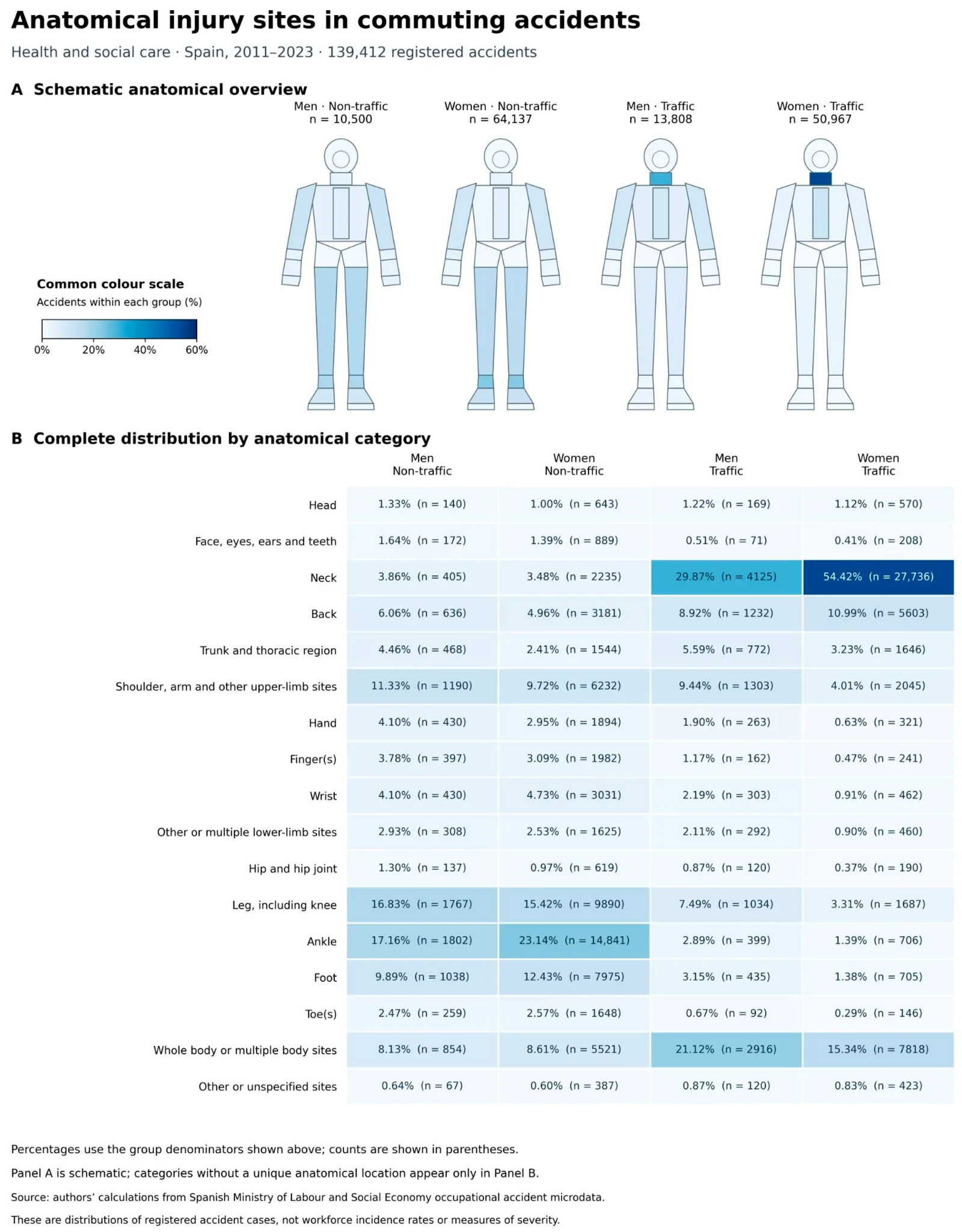
Anatomical injury-site distribution among commuting occupational accidents in health and social care activities, by sex and traffic involvement, Spain, 2011–2023. Panel (**A**) provides a schematic anatomical overview; Panel (**B**) presents all injury-site categories, with within-group percentages and absolute counts. Both panels use a common colour scale. Group denominators are shown above the silhouettes. Percentages describe registered accident cases, not workforce incidence rates or injury severity. Source: authors’ calculations from occupational accident microdata provided by the Spanish Ministry of Labour and Social Economy.

These differences in the anatomical distribution of injuries according to sex and traffic involvement are illustrated in Figure 3. In non-traffic-related accidents, lower-extremity injuries were particularly prominent among women, especially at the ankle, whereas traffic-related accidents showed a markedly different pattern, with neck injuries predominating among women. Among men, injuries were more widely distributed across different body regions, particularly in traffic-related accidents (Figure 3).

### 3.5. Adjusted Associations with Recorded Sickness Absence Duration

The expanded zero-truncated negative binomial model included all 139,412 accidents. The estimated NB2 dispersion parameter was 1.063 (95% CI: 1.054–1.073). The log likelihood was −657,854.00, AIC was 1,316,294.00, and BIC was 1,319,178.64. The equivalent ordinary negative binomial model had AIC 1,323,015.59. A zero-truncated Poisson model with the same covariates and age spline had AIC 6,714,743.58 and a Pearson statistic per residual degree of freedom of 60.59, compared with 1.38 for the zero-truncated NB2 model. Numerical optimisation converged and the observed information matrix was positive definite.

**Figure 4:**
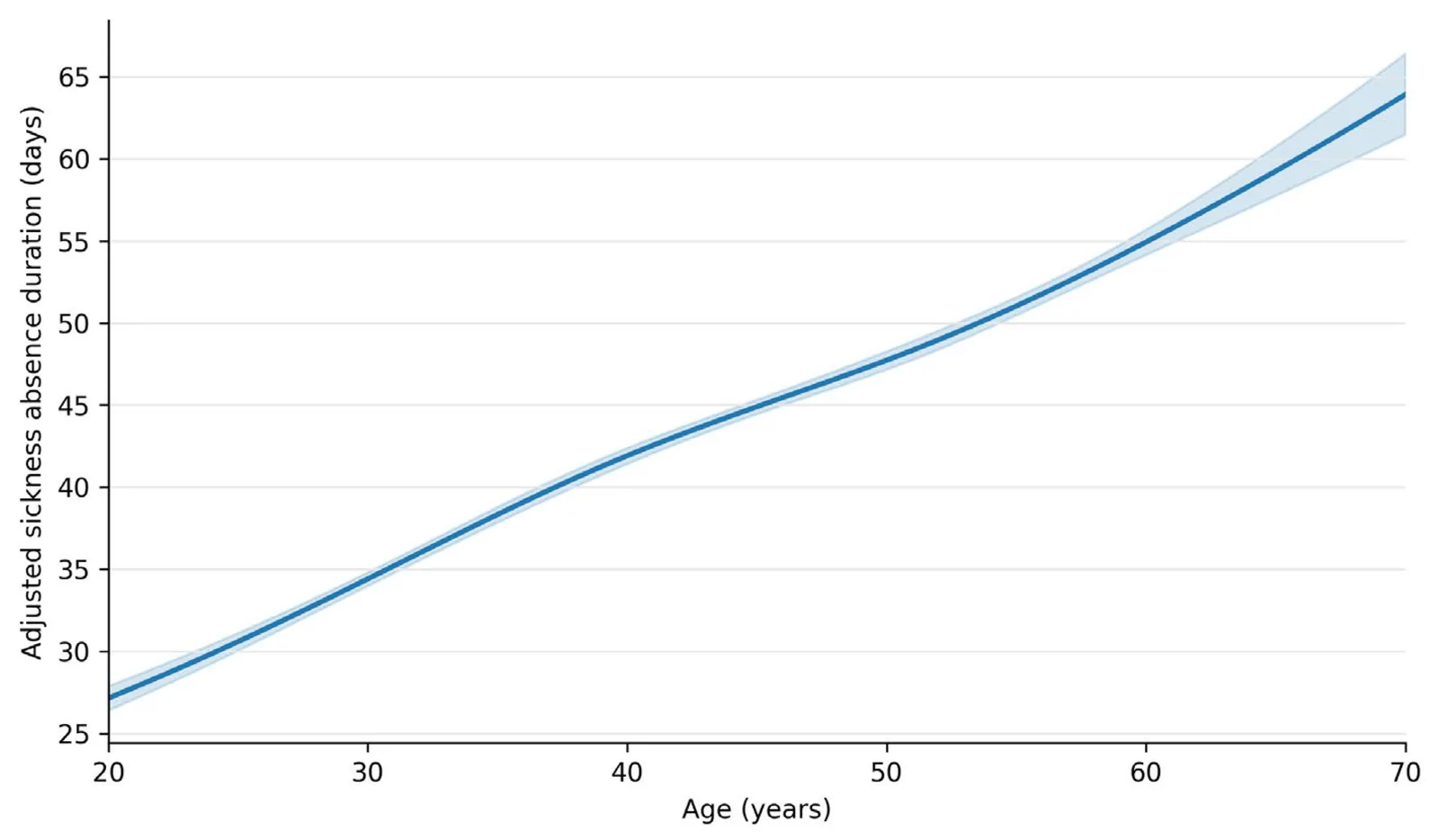
Adjusted mean recorded sickness absence duration by age among health and social care workers with commuting occupational accidents in Spain. Estimates were obtained from the expanded zero-truncated negative binomial model with a restricted cubic spline for age and marginally standardised over the observed distribution of the remaining covariates. The solid line represents the adjusted mean duration, and the shaded area indicates pointwise 95% confidence intervals.

Age was associated non-linearly with recorded sickness absence duration (robust Wald χ^2^ = 57.09, 2 degrees of freedom; *p* < 0.001). Adjusted mean durations at ages 25, 35, 45, 55, and 65 years were 30.59, 38.33, 44.90, 51.03, and 59.24 days, respectively (Table 6). Figure 4 illustrates this association across ages 20–70 years, showing a progressive increase in adjusted mean duration, with a steeper rise at older ages. The adjusted mean ratio for age 65 versus age 45 was 1.320 (95% CI: 1.281–1.360). These results do not support expressing the age association as a constant percentage increase per additional year.

In the primary expanded model, traffic-related accidents had a 27.1% longer adjusted mean duration than non-traffic-related accidents (mean ratio 1.271; 95% CI: 1.248–1.294). Return-journey accidents had a 10.2% longer adjusted mean duration than journeys to work (mean ratio 1.102; 95% CI: 1.087–1.117). The mean ratio for men versus women was 0.995 (95% CI: 0.977–1.014; *p* = 0.597). Shoulder, arm and other upper-limb sites and wrist injuries had adjusted mean ratios of 1.888 (95% CI: 1.751–2.037) and 1.773 (95% CI: 1.638–1.919), respectively, relative to head injuries (Table 7).

The year 2020 was associated with a higher adjusted mean duration than 2011 (mean ratio 1.205; 95% CI: 1.164–1.247). The corresponding ratio for 2019 was already 1.105 (95% CI: 1.070–1.142). These calendar-period associations should not be interpreted as causal effects of COVID-19 or mobility restrictions.

Excluding records at 545 days or fatal accidents, using broader occupational categories, and excluding the most influential 0.1% of records did not materially alter the principal age, traffic, or commuting-direction associations. Additional adjustment for injury type and severity attenuated the traffic mean ratio to 1.201 (95% CI: 1.180–1.222) and the return-journey ratio to 1.087 (95% CI: 1.073–1.101). In this additional model, men had a modestly shorter adjusted mean duration than women (mean ratio 0.959; 95% CI: 0.942–0.976). The absence of a sex association in the primary model was therefore not invariant to the adjustment set.

Observed-to-fitted mean-duration ratios across fitted-duration deciles ranged from 0.975 to 1.028. Randomized quantile residuals had a mean of 0.002 and a standard deviation of 0.965. However, the model did not fully reproduce the duration distribution, particularly the frequency of one-day records and parts of the upper tail. These discrepancies support cautious interpretation of distributional fit; inference was reported using sandwich standard errors, and sensitivity results are provided in the Supplementary Materials.

## 4. Discussion

This study provides a comprehensive analysis of commuting occupational accidents resulting in sickness absence among workers in the Spanish health and social care sector. Several findings deserve particular attention. First, women accounted for the large majority of recorded commuting accidents, consistent with the predominantly female composition of the sector. Sector-specific incidence rates were also higher among women in every age group examined in 2023. Second, the distribution of commuting accidents varied substantially with age: traffic-related accidents were relatively more concentrated among younger workers, whereas non-traffic-related accidents became increasingly prominent with age. Third, although accidents occurring on the way to work were considerably more frequent than those occurring on the way from work, return-journey accidents were associated with longer sickness absence. Fourth, traffic and non-traffic accidents produced markedly different anatomical injury patterns. Finally, the expanded multivariable analysis identified adjusted associations with age, traffic involvement, commuting direction, and anatomical injury site. No detectable sex difference was observed in the primary model, whereas a modest difference emerged after additional adjustment for injury type and recorded severity. These findings indicate that the factors associated with the occurrence and characteristics of commuting accidents are not necessarily the same as those determining their subsequent burden in terms of sickness absence.

The sex association depended on the adjustment set. The primary expanded model showed no detectable difference, whereas additional control for injury type and severity was associated with a modestly shorter duration among men. Traffic and return-journey associations also attenuated under clinical adjustment. These findings indicate that recorded injury characteristics contribute to the observed duration patterns and that sex should not be described as universally unrelated to sickness absence duration. Previous Spanish research has reported marked sex differences in commuting occupational accidents [1]. Nevertheless, the interpretation of this pattern requires consideration of exposure. The health and social care sector is highly feminised, and commuting exposure may also differ between men and women in terms of transport mode, distance, working schedules, occupational roles, and domestic responsibilities. The concept of “double presence”—the simultaneous demands of paid employment and domestic-family work—has been described as disproportionately affecting women and being associated with prolonged, rotating, and irregular working schedules [20]. These factors could contribute to differences in commuting conditions, although the present database does not contain information on family responsibilities, transport mode, or individual commuting exposure and therefore cannot directly test this explanation.

The revised analysis distinguishes accident incidence from recorded sickness absence duration among accident cases. After accounting for activity division, province, occupation, employment-contract code, source-file year, and accident characteristics, the duration association with age remained positive but was non-linear. Adjusted mean duration was 30.59 days at age 25, 44.90 days at age 45, and 59.24 days at age 65. Thus, the findings should not be reduced to a constant annual percentage increase, nor should recorded administrative duration be interpreted as verified recovery time. Older workers may have lower accident incidence because of differences in experience, behaviour, exposure, or work organisation, but may experience longer recovery after injury. This interpretation is consistent with previous evidence showing age-related reductions in work ability among healthcare professionals [11] and greater injury severity with increasing age among Spanish nursing workers [12]. From a preventive perspective, therefore, an ageing workforce does not necessarily imply a greater frequency of commuting accidents, but it may increase their consequences in terms of prolonged sickness absence.

Age was also strongly related to the type of commuting accident. Traffic-related accidents were concentrated disproportionately among younger workers, whereas the proportion of non-traffic-related accidents increased substantially with age. Differences in commuting behaviour may partly explain this pattern. Festini et al. reported age-related differences in commuting patterns among Italian nurses, including longer commuting distances among younger nurses [21]. Greater use of motorised transport or longer commuting distances could increase exposure to traffic-related events among younger workers, although transport mode and commuting distance were unavailable in our dataset. Conversely, the increasing relative importance of non-traffic accidents with age may reflect greater vulnerability to falls, slips, and similar events during commuting. Future studies incorporating transport mode, walking exposure, commuting distance, and environmental conditions would be needed to determine the mechanisms underlying this age gradient.

A second relevant finding concerns commuting direction. Approximately 70% of accidents occurred on the way to work, confirming the predominance of outbound commuting accidents previously reported in Spain [1]. However, return-journey accidents had longer mean recorded sickness absence in every reported age group and were associated with a 10.2% longer adjusted mean duration in the expanded primary model (mean ratio 1.102; 95% CI: 1.087–1.117). This apparent contrast between frequency and consequences is particularly noteworthy: accidents on the way to work were more common, whereas those occurring after work tended to have more prolonged consequences.

Several occupational mechanisms could contribute to this finding. Time pressure has been associated with cognitive failures and commuting near-accidents, suggesting that work-related psychosocial demands may extend beyond the workplace and affect commuting safety [22]. This may be particularly relevant to the journey to work, when punctuality requirements could contribute to time pressure. By contrast, the return journey occurs after occupational exposure and may therefore be influenced by accumulated fatigue and reduced alertness. A systematic review by Wagstaff and Sigstad Lie concluded that long working hours and shift or night work increase safety risks, with particular relevance to safety-critical sectors such as healthcare [23]. Among hospital nurses, Gold et al. found that rotating shift workers had twice the odds of nodding off while driving to or from work compared with nurses working only day or evening shifts [24]. Similarly, Barger et al. showed among medical interns that extended work shifts substantially increased the risk of motor vehicle crashes and that each additional extended shift increased the monthly risk of a crash specifically during the commute from work [25]. These studies provide possible explanations for commuting safety patterns, but evidence concerning accident occurrence does not establish why recorded absence was longer after return-journey accidents in this sample. Fatigue, shift work, sleep loss, journey duration, transport mode, and domestic responsibilities were not measured. Their contribution to the observed associations therefore remains hypothetical and cannot be inferred from commuting direction, sex, or traffic involvement alone.

Traffic involvement showed different crude and adjusted associations with recorded sickness absence duration. The overall crude mean was similar for traffic-related and non-traffic-related accidents (43.93 versus 44.35 days, respectively). However, traffic-related accidents had longer mean durations within every reported age group (Table 4). Traffic-related cases were more concentrated at younger ages and had a different anatomical injury-site distribution (Table 4 and Table 5). These compositional differences are consistent with the contrast between the crude overall comparison and the adjusted findings. In the expanded primary model, the marginally standardised adjusted mean ratio was 1.271 (95% CI: 1.248–1.294), indicating a 27.1% longer adjusted mean duration for traffic-related accidents. With additional adjustment for injury type and recorded severity, this ratio attenuated to 1.201 (95% CI: 1.180–1.222). The comparisons therefore depend on the characteristics accounted for in each model. They neither establish a causal effect of traffic involvement nor quantify the separate contribution of age or injury characteristics to the crude-adjusted difference. Longer recorded absence should not be equated with greater injury severity.

Previous research has identified occupational road traffic accidents as an important source of severe work-related injury [26]. The present findings extend this evidence by showing that traffic involvement remains associated with prolonged sickness absence even after controlling for several major worker and accident characteristics. In practical terms, traffic-related commuting accidents may be less frequent than some non-traffic events in particular groups but can impose a disproportionately high burden in terms of recovery and work absence. This distinction is important for prevention because interventions aimed solely at reducing accident frequency may overlook accident types associated with greater subsequent consequences.

The anatomical distribution of injuries provides further insight into the differences between traffic and non-traffic commuting accidents. Non-traffic accidents predominantly affected the lower extremities, especially the ankle, leg including the knee, and foot, whereas traffic-related accidents showed a markedly different pattern dominated by neck and multiple-site injuries. The contrast was particularly pronounced among women: neck injuries accounted for more than half of traffic-related accidents in women, while ankle injuries were the most common anatomical location in non-traffic accidents. These patterns are compatible with different injury mechanisms. Falls and slips during walking are likely to involve the lower extremities, whereas road traffic collisions expose occupants to acceleration–deceleration forces and more complex trauma affecting the neck and multiple body regions. The clear anatomical differences observed in our data reinforce the importance of analysing traffic and non-traffic commuting accidents separately rather than treating in itinere accidents as a homogeneous category.

Injury frequency did not necessarily correspond to the burden of recorded sickness absence. In the expanded primary model, shoulder, arm and other upper-limb injuries and wrist injuries had adjusted mean ratios of 1.888 (95% CI: 1.751–2.037) and 1.773 (95% CI: 1.638–1.919), respectively, relative to head injuries. These findings warrant consideration in relation to the manual and upper-limb demands of many healthcare and social care jobs. Tasks such as assisting or transferring patients, providing personal care, handling equipment, and performing repetitive or precise manual activities may be difficult when shoulder mobility, grip strength, or wrist function is impaired. Such limitations could contribute to prolonged absence even when an injury is not classified as clinically severe. However, individual task demands, functional capacity, rehabilitation, and workplace accommodations were not measured, and this explanation remains a possible interpretation rather than a mechanism demonstrated by the study. The findings support considering anatomical injury site when assessing absence-related burden and planning individual occupational health assessments, without assuming that a particular intervention would shorten absence.

The overall burden associated with these accidents was substantial. Mean sickness absence duration was 44.15 days, despite a median of only 25 days, reflecting the markedly right-skewed distribution and the presence of a smaller group of accidents associated with prolonged absence. Previous research based on Spanish occupational accident data reported mean sickness absence durations of 34.43 days among men and 32.91 days among women for commuting accidents [27]. Halla and Zweimüller, using Austrian administrative data, also demonstrated substantial and persistent employment consequences following commuting accidents [2]. Although differences in populations, periods, national compensation systems, and accident definitions limit direct comparisons, the relatively long duration observed in the present healthcare-sector sample underlines that commuting accidents represent not only a road or occupational safety issue but also an important source of work disability and organisational burden.

The findings support considering commuting accidents within occupational health management and assessing both their frequency and recorded absence-related burden. The associations with age, commuting direction, traffic involvement, and anatomical injury site may help identify priorities for further assessment, but they do not establish the effectiveness of specific preventive measures. Fatigue management, scheduling changes, safer travel arrangements, rehabilitation, and temporary task modifications are potential approaches informed by the wider literature and occupational practice, rather than interventions tested in this study. Their suitability should be assessed against actual worker needs, job demands, and commuting conditions, and their effects should be evaluated prospectively.

### Strengths and Limitations

The main strength of this study is the large population-based dataset, comprising 139,412 officially registered commuting occupational accidents over a 13-year period. The use of a national administrative register provides substantial statistical power and enables detailed analysis according to age, sex, commuting direction, traffic involvement, and injured body part. A further strength is the simultaneous consideration of accident incidence and sickness absence duration. These outcomes capture different dimensions of commuting occupational risk and revealed patterns that would have been obscured if only accident frequency had been examined.

Several limitations should also be considered. First, the study is retrospective and observational; consequently, the associations identified cannot be interpreted as causal. Second, the Spanish occupational accident database does not include several variables potentially relevant to commuting risk, including commuting distance and duration, mode of transport, detailed work schedules, shift length, night work, sleep duration, fatigue, psychosocial demands, family responsibilities, weather conditions, and road characteristics. Their absence limits the ability to explain some of the patterns observed, particularly differences by sex, age, and commuting direction.

Third, the database includes only officially reported occupational accidents resulting in at least one day of sickness absence. Minor commuting accidents without sickness absence are therefore not represented, and under-reporting may also occur [14]. Fourth, recorded sickness absence duration is not a direct measure of injury severity. Although injury type and recorded severity were included in an additional adjustment model, the data did not capture functional limitations, individual job demands, treatment and rehabilitation, administrative processes, or return-to-work arrangements. These factors may influence recorded duration and contribute to residual differences between accident groups. In addition, the interpretation of prolonged sickness absence is also limited by the upper boundary observed in the dataset. Records at 545 days could not be distinguished as completed absence episodes, administrative closures, or ongoing absences. Accordingly, the findings concern recorded sickness absence duration and should not be interpreted as estimates of time to recovery or verified return to work.

Although the zero-truncated NB2 model accommodates the positive outcome support and overdispersion, residual diagnostics showed departures from its assumed distribution, particularly among very short absences and in the upper tail. Sandwich standard errors and sensitivity analyses reduce reliance on the model-based variance assumption but do not establish perfect distributional fit or eliminate possible bias from a misspecified conditional mean. Sparse detailed occupation and contract categories also limit the precision of some nuisance estimates. Repeated accidents in the same worker could not be linked, so sandwich errors were calculated at accident level rather than clustered by worker. Detailed diagnosis codes were unavailable for 40,045 records (28.7%), although injury type and severity were available and examined in an additional adjustment model. Actual working schedules, fatigue, commuting exposure, and verified return-to-work status were not available. The 545-day boundary remained administratively ambiguous, and excluding its 34 observations was a sensitivity analysis rather than a time-to-return-to-work or censoring analysis.

Finally, workers who experienced multiple accidents during the study period could not be identified as repeated observations from the available anonymised dataset, and the individual accident was therefore treated as the unit of analysis.

Future research should combine occupational accident registers with information on work schedules, commuting distance, transport mode, sleep, fatigue, and psychosocial conditions. Prospective studies could determine whether the higher burden observed for return-journey accidents is related to accumulated fatigue, night work, or extended shifts. Further analyses should also examine interactions between age, traffic involvement, transport mode, and injured body part and evaluate whether preventive measures tailored to specific commuting profiles can reduce both accident occurrence and subsequent sickness absence.

## 5. Conclusions

Among registered commuting occupational accidents with sickness absence in the Spanish health and social care sector, older age, traffic involvement, return journeys, and anatomical injury site were associated with recorded absence duration after expanded adjustment. The age association was non-linear, and estimates for selected ages aid interpretation. The sex association depended on whether injury type and severity were additionally controlled. These results describe adjusted associations among accident cases and do not establish causal effects, verified recovery times, or an individually validated prediction model.

These findings support a broader approach to occupational safety in healthcare that extends beyond the physical workplace to include the journey to and from work. Prevention should consider not only the frequency of commuting accidents but also their potential consequences, with particular attention to older workers, traffic-related accidents, return journeys, and injuries associated with prolonged sickness absence.

## Figures and Tables

**Table 1:** Characteristics of commuting occupational accidents and sickness absence duration according to sex, commuting direction, traffic involvement, and injured body part.

Variable: Category	n (%)	Sickness Absence Duration (Days)	
		Median (P_25_–P_75_)	Mean (SD)
Sex:			
Male	24,308 (17.4)	25 (11–56)	45.84 (59.12)
Female	115,104 (82.6)	25 (11–53)	43.80 (55.269)
Commuting direction:			
on the way to work	97,799 (70.2)	24 (10–51)	42.64 (55.09)
on the way from work	41,613 (29.8)	28 (13–59)	47.70 (57.82)
Traffic involvement:			
Non-traffic-related accident	74,637 (53.5)	23 (10–54)	44.35 (58.02)
Traffic-related accident	64,775 (46.5)	27 (13–53)	43.93 (53.51)
Injured body part:			
Head	1522 (1.09)	19 (8–42)	38.89 (60.68)
Facial area	1340 (0.96)	13 (6–28)	25.9 (41.88)
Neck	34,501 (24.75)	27 (13–47)	37.13 (40.32)
Back	10,652 (7.64)	23 (10–45)	38.57 (49.06)
Torso and thoracic region	4430 (3.18)	27 (13–51)	44.18 (55.84)
Shoulder and arm	10,770 (7.73)	44 (16–98)	70.35 (75.36)
Hand	2908 (2.09)	26 (10–63)	46.52 (57.45)
Finger(s)	2782 (2.00)	26 (11–56)	42.75 (50.49)
Wrist	4226 (3.03)	43 (16–93)	65.01 (67.41)
Lower extremities, multiple sites	2685 (1.93)	23 (10–58)	49.85 (69.4)
Hip and hip joint	1066 (0.76)	22 (9–64)	55.07 (79.3)
Leg, including knee	14,378 (10.3)	22 (9–58)	48.54 (65.8)
Ankle	17,748 (12.7)	20 (10–41)	37.59 (50.48)
Foot	10,153 (7.28)	22 (10–51.5)	39.52 (48.64)
Toe(s)	2145 (1.54)	28 (12–52)	39.63 (42.46)
Multiple body sites	17,109 (12.27)	26 (11–56)	46.59 (61.3)
Unspecified	997 (0.72)	26 (11–56)	45.82 (58.21)

Note. n: absolute frequency; %: percentage; P_25_–P_75_: interquartile range; SD: standard deviation. Source: Authors’ calculations from occupational accident microdata provided by the Spanish Ministry of Labor and Social Economy, Spain, 2011–2023.

**Table 2:** Distribution of commuting occupational accidents and sickness absence duration by age group and sex.

Age Group (Years)	n (%)		Mean Recorded Sickness Absence Duration (Days)			
	Men	Women	Men	Women	t	*p*-Value
<30	4048 (16.65)	16,902 (14.68)	31.36	29.91	2.121	0.034
30–39	6385 (26.26)	24,658 (21.42)	38.66	36.82	2.841	0.005
40–49	6459 (26.57)	27,336 (23.74)	47.64	43.27	5.828	<0.001
50–59	5732 (23.58)	34,308 (29.8)	57.93	50.98	7.696	<0.001
≥60	1684 (6.92)	11,900 (10.33)	59.85	58.47	0.754	0.451

Note. n: absolute frequency; %: percentage (percentages are calculated within each sex group); t: Student’s *t*-test statistic; significance level (*p*-value). A *p*-value < 0.05 indicates a statistically significant difference in mean sickness absence duration between groups; therefore, the null hypothesis of equal mean sickness absence duration is rejected. Source: Authors’ calculations from occupational accident microdata provided by the Spanish Ministry of Labor and Social Economy, Spain, 2011–2023.

**Table 3:** Distribution of commuting occupational accidents and sickness absence duration by age group and commuting direction.

Age Group (Years)	n (%)		Mean Recorded Sickness Absence Duration (Days)			
	Journey to Work	Return Journey from Work	Journey to Work	Return Journey from Work	t	*p*-Value
<30	14,470 (14.79)	6480 (15.57)	28.66	33.59	−8.427	<0.001
30–39	21,562 (22.04)	9481 (22.78)	35.84	40.29	−7.844	<0.001
40–49	23,778 (24.31)	10,017 (24.07)	42.49	47.96	−8.477	<0.001
50–59	28,369 (29)	11,671 (28.04)	50.45	55.69	−7.523	<0.001
≥60	9620 (9.83)	3964 (9.52)	56.29	64.34	−6.060	<0.001

Note. n: absolute frequency; %: percentage (percentages are calculated within each commuting-direction group); t: Student’s *t*-test statistic; significance level (*p*-value). A *p*-value < 0.05 indicates a statistically significant difference in mean sickness absence duration between groups; therefore, the null hypothesis of equal mean sickness absence duration is rejected. Source: Authors’ calculations from occupational accident microdata provided by the Spanish Ministry of Labor and Social Economy, Spain, 2011–2023.

**Table 4:** Distribution of commuting occupational accidents and sickness absence duration by age group and traffic involvement.

Age Group (Years)	n (%)		Mean Recorded Sickness Absence Duration (Days)			
	Non-Traffic-Related	Traffic-Related	Non-Traffic-Related	Traffic-Related	t	*p*-Value
<30	6835 (9.15)	14,115 (21.79)	27.32	31.57	−7.370	<0.001
30–39	12,147 (16.27)	18,896 (29.17)	33.94	39.29	−10.016	<0.001
40–49	17,665 (23.66)	16,130 (24.9)	40.59	47.96	−12.511	<0.001
50–59	27,611 (36.99)	12,429 (19.18)	50.56	55.11	−6.638	<0.001
≥60	10,379 (13.9)	3205 (4.94)	57.59	62.03	−3.119	0.002

Note. n: absolute frequency; %: percentage (percentages are calculated within each traffic-involvement group); t: Student’s *t*-test statistic; significance level (*p*-value). A *p*-value < 0.05 indicates a statistically significant difference in mean sickness absence duration between groups; therefore, the null hypothesis of equal mean sickness absence duration is rejected. Source: Authors’ calculations from occupational accident microdata provided by the Spanish Ministry of Labor and Social Economy, Spain, 2011–2023.

**Table 5:** Distribution of injured body parts and mean sickness absence duration according to sex and traffic involvement.

Injured Body Part	Accidents n (%)				Mean Recorded Sickness Absence Duration (Days)			
	Non-Traffic-Related		Traffic-Related		Non-Traffic-Related		Traffic-Related	
	Men	Women	Men	Women	Men	Women	Men	Women
Head	140 (1.33)	643 (1)	169 (1.22)	570 (1.11)	32.94	32.84	53.06	42.99
Facial area	172 (1.63)	889 (1.38)	71 (0.51)	208 (0.4)	18.73	22.45	39.21	42.02
Neck	405 (3.85)	2235 (3.48)	4125 (29.87)	27,736 (54.41)	31.26	34.08	32.86	38.10
Back	636 (6.05)	3181 (4.95)	1232 (8.92)	5603 (10.99)	35.11	39.69	37.19	38.65
Torso and thoracic region	468 (4.45)	1544 (2.4)	772 (5.59)	1646 (3.22)	41.81	37.52	50.74	48.04
Shoulder and arm	1190 (11.33)	6232 (9.71)	1303 (9.43)	2045 (4.01)	66.68	75.98	66.00	58.13
Hand	430 (4.09)	1894 (2.95)	263 (1.9)	321 (0.62)	42.36	46.23	49.66	51.31
Finger(s)	397 (3.78)	1982 (3.09)	162 (1.17)	241 (0.47)	44.31	39.69	53.51	58.19
Wrist	430 (4.09)	3031 (4.72)	303 (2.19)	462 (0.9)	54.97	66.90	60.09	65.28
Lower extremities	308 (2.93)	1625 (2.53)	292 (2.11)	460 (0.9)	47.46	44.23	71.32	57.72
Hip and hip joint	137 (1.3)	619 (0.96)	120 (0.86)	190 (0.37)	56.87	51.47	72.79	54.35
Leg, including knee	1767 (16.82)	9890 (15.42)	1034 (7.48)	1687 (3.3)	47.75	45.33	66.63	57.18
Ankle	1802 (17.16)	14,841 (23.13)	399 (2.88)	706 (1.38)	31.97	37.04	59.80	51.06
Foot	1038 (9.88)	7975 (12.43)	435 (3.15)	705 (1.38)	34.14	38.74	54.71	47.03
Toe(s)	259 (2.46)	1648 (2.56)	92 (0.66)	146 (0.28)	35.42	38.80	44.85	53.25
Multiple body sites	854 (8.13)	5521 (8.6)	2916 (21.11)	7818 (15.33)	44.65	40.46	50.81	49.57
Unspecified	67 (0.63)	387 (0.6)	120 (0.86)	423 (0.82)	45.27	43.14	53.10	46.29

Note: Percentages describe the anatomical injury-site distribution within each sex and traffic-involvement group. Denominators are 10,500, 64,137, 13,808, and 50,967, respectively. Mean durations are unadjusted. Source: Authors’ calculations from occupational accident microdata provided by the Spanish Ministry of Labor and Social Economy, Spain, 2011–2023.

**Table 6:** Adjusted mean recorded sickness absence duration at selected ages in the expanded zero-truncated NB2 model.

Age	Adjusted Mean Days	95% CI	Ratio vs. Age 45
25	30.59	30.06–31.14	0.681
35	38.33	37.86–38.81	0.854
45	44.90	44.46–45.34	1.000
55	51.03	50.48–51.58	1.137
65	59.24	57.76–60.76	1.320

Note. All other covariates retain their observed values; the conditional means are averaged across the study sample. These are adjusted associations, not causal effects or externally validated individual predictions. Source: Authors’ calculations from occupational accident microdata provided by the Spanish Ministry of Labour and Social Economy, Spain, 2011–2023.

**Table 7:** Marginally standardised adjusted mean ratios of recorded sickness absence duration following commuting occupational accidents in the expanded zero-truncated NB2 model.

Contrast	Mean Ratio	95% CI	*p*-Value
Men vs. women	0.995	0.977–1.014	0.597
Traffic vs. non-traffic	1.271	1.248–1.294	<0.001
Return vs. outward journey	1.102	1.087–1.117	<0.001
Face, eyes, ears and teeth vs. head	0.713	0.636–0.799	<0.001
Neck vs. head	0.953	0.885–1.025	0.196
Back vs. head	1.032	0.956–1.114	0.418
Trunk and thoracic region vs. head	1.125	1.037–1.220	0.005
Shoulder, arm and other upper-limb sites vs. head	1.888	1.751–2.037	<0.001
Hand vs. head	1.338	1.228–1.457	<0.001
Finger(s) vs. head	1.213	1.115–1.320	<0.001
Wrist vs. head	1.773	1.638–1.919	<0.001
Other or multiple lower-limb sites vs. head	1.404	1.284–1.535	<0.001
Hip and hip joint vs. head	1.446	1.295–1.616	<0.001
Leg, including knee vs. head	1.365	1.265–1.473	<0.001
Ankle vs. head	1.094	1.015–1.180	0.020
Foot vs. head	1.161	1.075–1.254	<0.001
Toe(s) vs. head	1.151	1.056–1.253	0.001
Whole body or multiple body sites vs. head	1.204	1.117–1.298	<0.001
Other or unspecified sites vs. head	1.169	1.051–1.300	0.004

Note. Ratios are calculated from positive-duration conditional means averaged over the empirical distribution of the remaining covariates; they are not Exp(B). All models adjust for activity division, workplace province, detailed occupation, source-file year and employment-contract code, in addition to the original covariates. Age is represented by a four-knot restricted cubic spline. Confidence intervals use the observation-level sandwich covariance estimator and delta method. Head is the injury-site reference. Age estimates are presented in Table 6. Source: Authors’ calculations from occupational accident microdata provided by the Spanish Ministry of Labour and Social Economy, Spain, 2011–2023.

## Data Availability

The data analysed in this study were obtained from the Statistics on Accidents at Work (Estadística de Accidentes de Trabajo), compiled by the Spanish Ministry of Labour and Social Economy (Ministerio de Trabajo y Economía Social) from occupational accident reports registered through the Delt@ system. The microdata are not publicly available for direct download. However, they may be requested from the Spanish Ministry of Labour and Social Economy for research purposes. Researchers requesting access are required to undertake to use the data exclusively for research purposes and not for commercial purposes. Access to the data is subject to the conditions and authorisation established by the Ministry.

## Supplementary Materials

The following supporting information can be downloaded at https://www.mdpi.com/article/10.3390/healthcare14213709/s1, Tables S1–S3 present model comparisons, sensitivity analyses, and distributional diagnostics; Figure S1 presents diagnostic plots; Table S4 documents variable coding and analytical decisions; Table S5 reports sequential sample selection and missing information; Table S6 summarizes selected additional covariates; and Table S7 provides the completed STROBE reporting checklist.

## Abbreviations

The following abbreviations are used in this manuscript:

CNAE	Spanish National Classification of Economic Activities (Clasificación Nacional de Actividades Económicas)
CI	Confidence Interval
Delt@	Electronic Declaration of Injured Workers
NACE	Statistical Classification of Economic Activities in the European Community
SD	Standard Deviation

## Appendix A. Coding of Traffic Involvement and Anatomical Injury Sites

### Appendix A.1. Traffic Involvement

Traffic involvement was identified using the Accidente de tráfico field of the Spanish Delt@ occupational accident report. This field records whether the occupational accident resulted from a traffic accident (yes/no), according to the official reporting instructions [15]. Records were classified as traffic-related or non-traffic-related using this indicator.

Traffic involvement and commuting status represent separate characteristics. Commuting status identifies accidents occurring during the journey to or from work, whereas traffic involvement identifies whether the event was recorded as a traffic accident. Consequently, commuting accidents include both traffic-related and non-traffic-related events.

**Table A1:** Definition of traffic-involvement categories.

Category Used in the Study	Value of the Delt@ Traffic Indicator
Traffic-related accident	Yes
Non-traffic-related accident	No

### Appendix A.2. Anatomical Injury Sites

Anatomical injury site was obtained from the Parte del cuerpo lesionada field of the Delt@ occupational accident report. This field identifies the anatomical location affected using the official two-digit classification provided in Annex II, Table 7 of the Delt@ completion guide [15]. Anatomical location is distinct from injury type: it describes where the injury occurred rather than whether it was a fracture, sprain, wound, or another type of injury.

Table A2 summarises the original classification. Codes identifying unspecified locations, other locations, systemic effects, and multiple affected sites have distinct meanings and should not be interpreted as interchangeable.

**Table A2:** Overview of the original Delt@ anatomical injury-site classification.

Anatomical Classification	Original Codes
Head	10, 11, 18, 19
Face, eyes, ears and teeth	12, 13, 14, 15
Neck	20, 21, 29
Back	30, 31, 39
Trunk and thoracic region	40, 41, 42, 43, 48, 49
Shoulder, arm and other upper-limb sites	50, 51, 52, 58, 59
Hand	53
Finger(s)	54
Wrist	55
Other or multiple lower-limb sites	60, 68, 69
Hip and hip joint	61
Leg, including knee	62
Ankle	63
Foot	64
Toe(s)	65
Whole body or multiple body sites	70, 71, 78
Other or unspecified sites	00, 99

Source: Delt@ occupational accident report completion guide, Annex II, Table 7 [15].

For the analyses presented in this article, anatomical injury sites were consolidated into the categories reported in Table 1, Table 5 and Table 7. The unspecified category was retained in the analyses and did not constitute an exclusion criterion. No records were excluded because of missing data.
