# Supplementary material for: Commuting Accidents and Sickness Absence Among Health and Social Care Workers in Spain: The Role of Age and Accident Characteristics
Source: Healthcare (Basel). 2026 Nov 1;14(21):3709. doi: 10.3390/healthcare14213709 (PMC13763373; doi:10.3390/healthcare14213709)
Supplement: Supplementary file 1 [file healthcare-14-03709-s001.zip › healthcare-4594411-supplementary.pdf]

Supplementary materials

Table S1. Model comparison using the same 139,412 observations and the same expanded adjustment set.

Model	α	Log likelihood	AIC	BIC
Expanded NB, linear age	0.923	-661,254.99	1,323,091.99	1,325,956.94
Expanded ZTNB, linear age	1.064	-657,892.96	1,316,367.91	1,319,232.86
Expanded ZTNB, spline age	1.063	-657,854.00	1,316,294.00	1,319,178.64
Expanded NB, spline age	0.923	-661,214.80	1,323,015.59	1,325,900.23

The spline model has three age coefficients, compared with one in the linear model. For the primary ZTNB model, 292 regression coefficients plus one dispersion parameter were estimated. The clinical adjustment model has 336 parameters in total. The zero-truncated Poisson comparator has 292 regression parameters and AIC 6,714,743.58. AIC and BIC are not used to rank sensitivities fitted to different subsets.

Table S2. Sensitivity analyses for the principal adjusted mean ratios.

Model	Men vs women	Traffic	Return	Age 65 vs 45
Expanded ZTNB, spline age	0.995	1.271	1.102	1.320
Expanded NB, spline age	0.995	1.270	1.101	1.319
Exclude 34 cases at 545 days	0.994	1.272	1.102	1.315
Exclude 117 fatal accidents	0.997	1.272	1.102	1.320
Add injury type and severity	0.959	1.201	1.087	1.208
Use accident year ANOAC	0.995	1.271	1.102	1.319
Use broad occupational groups	0.995	1.271	1.100	1.312
Exclude top 0.1% influence	0.997	1.275	1.104	1.324

The influence sensitivity excluded 140 observations (0.1004%) and retained 139,272. Excluding 545-day cases retained 139,378; excluding fatal cases retained 139,295. No censoring status was imputed.

Table S3. Distributional fit of the primary model.

Duration in days	Observed records	Expected records
1	91	3924.2
2–7	21371	20100.0
8–14	22124	18760.6
15–30	36872	30886.0
31–60	28743	32171.9
61–90	12688	15573.0

Duration in days	Observed records	Expected records
91–180	12543	14562.5
181–365	4655	3212.9
366–545	325	195.3
546–inf	0	25.7

The model predicts approximately 3,924 one-day records, compared with 91 observed. It also has a nonzero theoretical tail beyond 545 days (approximately 25.7 expected records). These are diagnostic probabilities, not additional observed accidents. Mean calibration by decile is close, but this does not establish complete distributional fit.

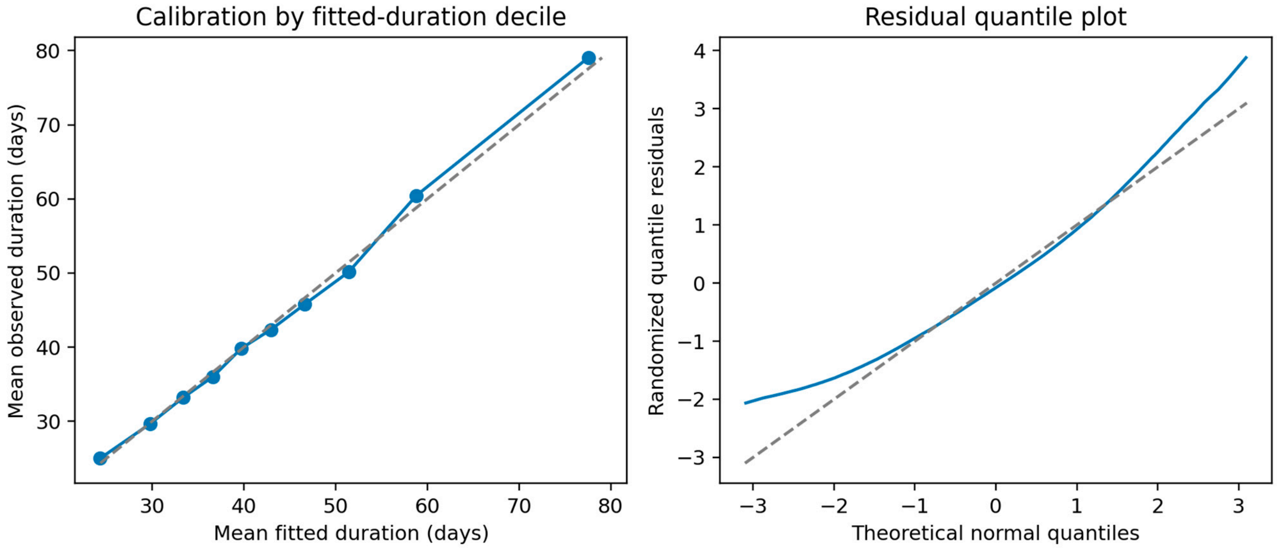

Figure S1. Calibration by fitted-duration decile and randomized quantile residual plot for the expanded zero-truncated NB2 model.

Variable coding and analytical decisions

Table S4. Source fields and coding used for the revised regression analyses. Administrative codes are retained as recorded unless a recoding rule is explicitly stated. Reference categories refer to the actual model implementation.

Variable	Field	Coding and treatment
Recorded absence	DIASBAJA	Integer days; observed range 1–545. Positive duration required. No analytical upper cap. All 139,412 records used; 34 at 545 days excluded only in sensitivity analysis.
Age	EDAD	Continuous years; four-knot restricted cubic spline at 24, 39, 51 and 62. Internal basis uses (age − 40)/10. Descriptive groups: <30, 30–39, 40–49, 50–59, ≥60. Adjusted ages: 25, 35, 45, 55, 65; contrast reference 45.
Sex	SEXO	1 = men; 2 = women (reference).
Traffic involvement	ACCTRAF	0 = non-traffic (reference); 1 = traffic. This indicator does not identify transport mode.

Variable	Field	Coding and treatment
Commuting direction	HORATRABIDA0VUELTA99	Derived from Hora de trabajo / HORATRAB: 0 = journey to work (reference); 99 = return journey. Codes denote direction, not actual hours worked.
Anatomical injury site	PCLESION; GrLesiónNacho	Original two-digit injury-site codes mapped to 17 categories in Table A2. Group codes in GrLesiónNacho: 10, 12, 20, 30, 40, 50, 53, 54, 55, 60, 61, 62, 63, 64, 65, 70, 99. Reference = 10 (head). Other/unspecified group retained.
Activity division	CNAE2digitos	86 = human health (reference); 87 = residential care; 88 = social work without accommodation. Categorical.
Workplace geography	PROVCEN	52 observed province codes; categorical, without regional aggregation. Reference = 28. This is workplace province, not residence or accident location.
Occupation	CNO2011	161 observed three-digit codes; categorical, no primary regrouping. Reference = 561. Sensitivity: broad group = integer quotient CNO2011/100.
Reporting period	YEAR	13 annual accident year (AÑO), 2011–2023; categorical. Reference = 2011. No separate COVID-19 indicator or causal pandemic contrast.
Contract	TIPCONT	45 categories after recoding. Missing values in 78 records become −1 (unknown); recorded code 0 retained separately. Reference = 100. Other codes kept as distinct categories.
Injury type	DLESION	Additional adjustment only; 41 observed codes retained categorically; reference = 32. Recorded unspecified codes retained; no diagnoses inferred from these codes.
Recorded severity	GLESION	Additional adjustment only; four recorded categories; reference = 1. Fatal category = 4; excluded only in nonfatal sensitivity. Codes 2 and 3 remain distinct.
Detailed diagnosis	DIAG	40,045 records lack usable information. Not included in the primary or additional injury-type/severity model; no imputation.
Record selection	ACCLUG; TIPOACC	Analytical extract contains ACCLUG = 3 (commuting), TIPOACC = 1 (accident). Relapse selection occurred before the supplied analytical extract; upstream counts are reported by the authors in Section 2.2.

Model interpretation. NB2 latent variance = $\mu + \alpha\mu^2$; the primary likelihood is conditional on $Y > 0$. Positive-duration means are $\mu/[1 - P(Y = 0)]$. Reported ratios compare sample-averaged conditional means, not Exp(B). Confidence intervals use accident-level sandwich covariance and the delta method. No verified return-to-work event or censoring indicator was reconstructed.

Sample selection and missing information

Table S5. Sequential selection counts. Counts before the analytical extract are reported in Section 2.2; the final extract and absence of analytical row exclusions were checked by the reanalysis code.

Step	Retained	Removed at this step
All-sector accident and relapse records	7,695,970	Not applicable
Restrict to activity divisions 86–88	746,556	6,949,414 outside selected divisions
Restrict to commuting records	144,938	601,618 outside commuting category
Exclude relapses	139,412	5,526 relapses

Step	Retained	Removed at this step
Check commuting-direction codes	139,412	0
Address missing primary-model fields	139,412	0; 78 unknown contracts retained
Primary regression	139,412	0

Missingness. Outcome, age, sex, traffic involvement, commuting direction, injury-site group, activity division, province, occupation and accident year (AÑO) had no missing values in the analytical extract. Contract information was missing in 78 records (0.056%); these records were retained as a separate category. Injury type and recorded severity had no missing values. Detailed diagnosis was unavailable for 40,045 records (28.725%) and was not modelled. Recorded unspecified codes are distinct from missing values and were retained.

Table S6. Distribution of selected additional categorical covariates. Coding decisions and reference categories are documented in Table S4.

Field	Code	Records	%
CNAE2digitos	86	90,168	64.68
CNAE2digitos	87	24,544	17.61
CNAE2digitos	88	24,700	17.72
YEAR	2011	8,641	6.20
YEAR	2012	8,959	6.43
YEAR	2013	9,770	7.01
YEAR	2014	9,951	7.14
YEAR	2015	10,400	7.46
YEAR	2016	11,173	8.01
YEAR	2017	11,585	8.31
YEAR	2018	11,357	8.15
YEAR	2019	11,263	8.08
YEAR	2020	9,505	6.82
YEAR	2021	12,529	8.99
YEAR	2022	11,504	8.25
YEAR	2023	12,775	9.16
GLESION	1	137,502	98.63
GLESION	2	1,715	1.23
GLESION	3	78	0.06
GLESION	4	117	0.08

STROBE reporting checklist

Table S7. Completed STROBE checklist applied to the retrospective administrative-record analysis. The cross-sectional checklist is the closest applicable framework because the analysis uses accident records without a reconstructed longitudinal return-to-work follow-up. The unit of analysis is an accident, not a uniquely identified worker. Locations refer to manuscript sections and table identifiers, which remain stable when pagination changes. Recommendation labels below are abbreviated and paraphrased; this table is a completed reporting crosswalk, not the official blank form.

Source: STROBE Initiative, checklist for cross-sectional studies. https://www.strobe-statement.org/checklists/ (accessed on 12 October 2026).

Title and introduction

Item	Reporting topic	Location and response
1a	Design identification	Abstract Methods; Section 2.1. Retrospective observational secondary administrative-data analysis.
1b	Balanced abstract	Abstract. Population, methods, principal estimates and conditional interpretation of sex association reported.
2	Rationale	Introduction. Background, knowledge gap and rationale for analysis of registered commuting accidents.
3	Objectives	Introduction final paragraph; Abstract Background/Objectives. Descriptive characterisation and adjusted associations; no prespecified causal hypotheses asserted.

Methods

Item	Reporting topic	Location and response
4	Design	Section 2.1. Administrative record analysis, accident-level unit; no reconstructed follow-up.
5	Setting and dates	Sections 2.1–2.2; Table S5. Spain, divisions 86–88, annual files 2011–2023. Extraction date and episode-update history unavailable and acknowledged.
6a	Eligibility and selection	Section 2.2; Table S5. Registered commuting accidents with positive absence; division restriction and relapse exclusions. No participant recruitment.
7	Variable definitions	Section 2.3; Appendix A; Table S4. Outcome, focal covariates, additional adjustment fields, coding and injury-type/severity sensitivity.
8	Data measurement	Sections 2.2–2.3; Appendix A; Table S4. Same administrative reporting system across comparison groups; episode endpoint and diagnostic limitations acknowledged. No assumption of verified recovery.
9	Bias	Sections 2.4 and 4.1. Expanded adjustment, flexible age term, robust inference and sensitivity checks; reporting, selection, residual confounding and endpoint uncertainties discussed.
10	Study size	Section 2.2; Table S5. All eligible records in supplied annual files; no sampling or power-based selection.
11	Quantitative variables	Section 2.4; Tables 2–4, 7 and S4. Continuous age spline with explicit knots, descriptive age groups, positive integer duration.
12a	Statistical methods	Section 2.4. Descriptive and bivariate tests, NB2 likelihoods, expanded adjustment, marginal standardisation, sandwich covariance and delta method.
12b	Subgroups and interactions	Section 2.4; Tables 2–5. Descriptive stratification by age, sex, traffic and direction. No formal interaction model reported; proposed for future research.

Item	Reporting topic	Location and response
12c	Missing data	Sections 2.4 and 4.1; Tables S4–S5. 78 missing contracts retained as unknown; 40,045 unusable diagnoses excluded from covariate set, not case sample. No imputation.
12d	Sampling methods	Not applicable. Entire eligible supplied extract analysed; no survey sampling or weights.
12e	Sensitivity analyses	Section 2.4 and 3.5; Table S2. Ordinary NB2, endpoint exclusion, nonfatal cases, clinical adjustment, accident year, broad occupation and influence exclusion.

Results

Item	Reporting topic	Location and response
13a	Selection numbers	Section 2.2 and 3.1; Table S5. Sequential counts from 7,695,970 records to 139,412 accidents; all included in primary model.
13b	Exclusion reasons	Section 2.2; Table S5. Outside divisions, noncommuting records and relapses; no direction- or missingness-based case exclusions.
13c	Selection diagram	Sequential selection table supplied as Table S5. A graphical flow diagram was considered; the table provides the same ordered counts and reasons.
14a	Descriptive characteristics	Sections 3.1–3.4; Tables 1–5 and S6.
14b	Missingness by variable	Section 2.4; notes accompanying Table S5.
15	Outcome summaries	Section 3.1; Table 1 and S3. Mean, SD, median, quartiles, range, endpoint count and distributional frequencies.
16a	Crude and adjusted estimates	Tables 1–5 provide crude duration summaries; Tables 6–7 provide adjusted mean ratios/means with 95% CIs. Section 2.4 lists adjustment variables and rationale. No claim that all crude comparisons have confidence intervals.
16b	Category boundaries	Tables 2–4 and S4. Age groups <30, 30–39, 40–49, 50–59, ≥60; spline knots explicit in Methods.
16c	Absolute interpretation	Absolute risk conversion not applicable: the model concerns duration among accident cases. Table 7 provides adjusted absolute mean durations in days.
17	Additional analyses	Section 3.3–3.5; Tables 2–5 and S1–S3; Figure S1. Stratified summaries, model comparison, diagnostics and sensitivities.

Discussion and funding

Item	Reporting topic	Location and response
18	Main findings	Discussion opening; Conclusions. Nonlinear age, traffic, direction and injury-site associations; adjustment-dependent sex estimate.
19	Limitations	Section 4.1. Unmeasured factors, administrative endpoint, reporting coverage, distributional misspecification, sparse categories and inability to link repeated accidents. Direction/magnitude of residual bias cannot be quantified.
20	Cautious interpretation	Discussion and Conclusions; Section 4.1. Associational interpretation, possible mechanisms, conditional preventive implications and exploratory multiplicity caution.
21	Generalisability	Section 4.1. Restricted to covered registered positive-duration accident cases in selected Spanish activities; no whole-workforce accident-risk inference.

Item	Reporting topic	Location and response
22	Funding	Funding statement. No external funding for this research. Ministry identified as administrative data provider in Methods and Acknowledgments; financing of the underlying reporting system was not evaluated.
